# The Origin and Evolution of Plant Flavonoid Metabolism

**DOI:** 10.3389/fpls.2019.00943

**Published:** 2019-08-02

**Authors:** Keiko Yonekura-Sakakibara, Yasuhiro Higashi, Ryo Nakabayashi

**Affiliations:** RIKEN Center for Sustainable Resource Science, Yokohama, Japan

**Keywords:** secondary metabolites, flavonoid, polyketide synthase, 2-oxoglutarate-dependent dioxygenase, cytochrome P450, short-chain dehydrogenase/reductase, plant

## Abstract

During their evolution, plants have acquired the ability to produce a huge variety of compounds. Unlike the specialized metabolites that accumulate in limited numbers of species, flavonoids are widely distributed in the plant kingdom. Therefore, a detailed analysis of flavonoid metabolism in genomics and metabolomics is an ideal way to investigate how plants have developed their unique metabolic pathways during the process of evolution. More comprehensive and precise metabolite profiling integrated with genomic information are helpful to emerge unexpected gene functions and/or pathways. The distribution of flavonoids and their biosynthetic genes in the plant kingdom suggests that flavonoid biosynthetic pathways evolved through a series of steps. The enzymes that form the flavonoid scaffold structures probably first appeared by recruitment of enzymes from primary metabolic pathways, and later, enzymes that belong to superfamilies such as 2-oxoglutarate-dependent dioxygenase, cytochrome P450, and short-chain dehydrogenase/reductase modified and varied the structures. It is widely accepted that the first two enzymes in flavonoid biosynthesis, chalcone synthase, and chalcone isomerase, were derived from common ancestors with enzymes in lipid metabolism. Later enzymes acquired their function by gene duplication and the subsequent acquisition of new functions. In this review, we describe the recent progress in metabolomics technologies for flavonoids and the evolution of flavonoid skeleton biosynthetic enzymes to understand the complicate evolutionary traits of flavonoid metabolism in plant kingdom.

## Introduction

Plants have the ability to produce a huge variety of metabolites. Over 1,000,000 metabolites are predicted to be present in the entire plant kingdom (Afendi et al., 2012). Most of these are secondary metabolites (also referred to as specialized metabolites) that play a wide range of physiological and ecological roles including defense against herbivores and pathogens, attractants for pollinators and seed carriers, and signaling. During the long process of evolution, plants have gained, expanded, and sometimes lost their capabilities to produce this huge array of metabolites, which provides the adaptive mechanisms needed for survival in changing environments.

Flavonoids form one of the major groups of specialized metabolites, and include over 9,000 compounds (Williams and Grayer, 2004; Anderson and Markham, 2006). According to Nomenclature of flavonoids (IUPAC Recommendations, 2017), the term “flavonoid” is applied to (1) compounds structurally based on derivatives of a phenyl-substituted propylbenzene having a C15 skeleton, (2) compounds with a C16 skeleton that are phenyl-substituted propylbenzene derivatives (rotenoids), (3) flavonolignans based on derivatives of phenyl-substituted propylbenzene condensed with C_6_-C_3_ lignan precursors (Rauter et al., 2018). In a restricted sense, the term “flavonoid” is used only for those compounds with a C_6_-C_3_-C_6_ carbon framework exhibiting the structure of a chromane or that of a chromene such as flavans, flavones, flavonols, and anthocyanidins. Chalcones, dihydrochalcones, and aurones are flavonoids in a broad sense, but not in a limited sense.

Flavonoids, including chalcones, flavones, flavonols, anthocyanins, and proanthocyanidins, are widely distributed in the plant kingdom, and their metabolic pathways have been extensively studied using both biochemical and molecular biological techniques. Until recently, it was believed that liverworts and mosses were the oldest flavonoid-producing plants (Rausher, 2006; Bowman et al., 2017). The genes encoding enzymes in the phenylpropanoid biosynthetic pathway, including the first two enzymes for flavonoid biosynthesis (chalcone synthase and chalcone isomerase) had not been found in the algal genera *Chlamydomonas, Micromonas, Ostreococcus*, and *Klebsormidium*, although genes encoding enzymes in the shikimate pathway were found in algae, liverworts, mosses, lycophytes, ferns and horsetails, gymnosperms, and angiosperms (Bowman et al., 2017). However, flavones, isoflavones, and flavonols were detected in microalgae from five different evolutionary lineages (*Cyanobacteria, Rhodophyta, Chlorophyta, Haptophyta*, and *Ochrophyta*) using ultra-high performance liquid chromatography with tandem mass spectrometry (Goiris et al., 2014). This suggests that plants may have acquired the ability to produce flavonoids earlier than we previously thought. Furthermore, in extant plants flavonoids play important roles as ultraviolet-B (UV-B) protectants, pigments that attract pollinators, phytoalexins, signaling molecules, and regulators of auxin transport and fertility (Gould and Lister, 2006). It has been proposed that defense against UV irradiation and regulation of plant hormone action were the original functions of flavonoids in the earliest flavonoid producing plants (Stafford, 1991; Shirley, 1996; Rausher, 2006). These functions have been considerably diversified during plant evolution. Thus, the study of flavonoids is a useful approach to understand how plants acquired the ability to produce specialized metabolites, and then build the metabolic pathways needed to produce such a huge variety of metabolites during the course of their evolution. This study will shed light on the relationships between genes/proteins and metabolites, and between the metabolites and their physiological functions. In this review, we describe the structural diversity of flavonoids distributed in the plant kingdom, and how plants acquired flavonoid biosynthetic genes during their evolution.

## Metabolomics Sheds Light on the Evolution of Flavonoids

### Distribution of Flavonoids in the Plant Kingdom

**Figure 1:**
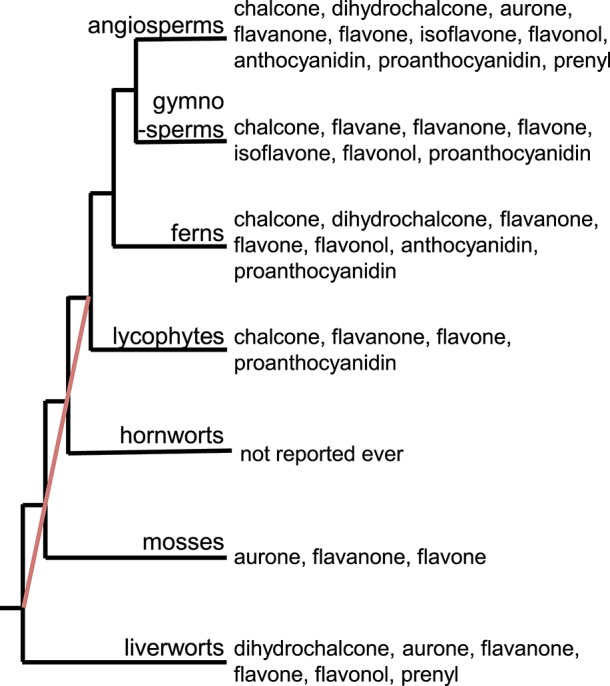
Distribution of flavonoid subclasses in the plant kingdom. The red line indicates the phylogenetic relationships in the bryophyte lineages that are unresolved, as stated in Bowman et al. (2017).

It is estimated that there are over 9,000 flavonoids in the plant kingdom (Williams and Grayer, 2004; Anderson and Markham, 2006). Research on flavonoids has shown that they are distributed across the plant kingdom, including angiosperms, gymnosperms, and pteridophytes (Harborne, 1988; Tohge et al., 2013). The abundance of information about flavonoids in different species allows us to identify which flavonoid subclasses (e.g., chalcones, flavones, flavonols, anthocyanins, and proanthocyanidins) are found in each subgroup of plants (Figure 1). Flavone and flavanone are found in all plant groups except for hornworts. To our knowledge, no flavonoids have been reported in hornworts. As plant groups have evolved and diversified, so have the flavonoid subclasses produced within each group. For example, the flavonoid aglycones are most diversified in the angiosperms. In addition to flavanones and flavones, chalcones, flavonols, and proanthocyanidins are found in multiple groups. Interestingly, the prenylflavonoids are found in both liverworts and angiosperms: while more than 1,000 prenylflavonoids have been isolated from legumes (Yazaki et al., 2009), prenyldihydrochalcone has been found in the liverworts *Radula variabilis* and *Radula* spp. (Asakawa et al., 1978, 1982). These data suggest that the two groups of plants gained the ability to produce prenylflavonoids independently, or that many groups have lost the ability to produce prenylflavonoids during their evolution. Flavonoid molecules provide concrete evidence for the existence of the corresponding flavonoid biosynthetic genes in the plants. Analytical approaches for identifying flavonoids is therefore important to understand the evolution of flavonoid metabolism in the plant kingdom.

### Cutting-Edge Metabolomics Technologies Contribute to Understanding the Evolution of Flavonoid Metabolism

So far, chromatographic and spectroscopic approaches have been used to analyze the structures of flavonoids and to reveal their chemical diversity. Previously, paper chromatography, thin layer chromatography, column chromatography, and liquid chromatography (LC) were the main techniques used to study flavonoids. Crude extracts containing flavonoids are obtained through liquid-liquid partitioning. For instance, flavonoid aglycones and their mono-glycosides can be mainly extracted in ethylacetate, whereas the flavonoid di- or tri-glycosides are extracted in *n*-butanol. Chromatographic techniques are then used to purify the flavonoids from the ethylacetate or *n*-butanol fractions, in several steps. Finally, the isolated flavonoids are analyzed by nuclear magnetic resonance and mass spectrometry to elucidate their structures. This is an unequivocal and straightforward approach to identifying flavonoids, however, it is time-consuming.

With the development of metabolomics technologies, flavonoids can now be analyzed much more accurately and precisely than before. LC-tandem mass spectrometry (LC-MS/MS) has become the preferred approach for analyzing flavonoids. It had been considered that there was no flavonoids in algae. However, identification of flavonoids such as intermediates and end products by an LC-MS/MS approach proved the existence of the flavonoid biosynthetic ability in microalgae (Goiris et al., 2014). It suggests that cutting-edge metabolomics technologies in every single plant species can unveil the flavonoids that have been missed.

The integration of LC-MS/MS with cheminformatics approaches provides a powerful tool for surveying flavonoid diversity in a high-throughput way (Tsugawa et al., 2016; Akimoto et al., 2017). Glycosylated, acylated, and prenylated flavonoid molecules and their aglycones can be separated using simple combinations of solvents and LC columns. The separated molecules are then ionized for MS/MS analysis. The product ions are derived from their precursors through the cleavage of ether and ester bonds or even the prenyl moiety. These simple fragmentation steps allow us to estimate the structures of the flavonoids. Cheminformatic tools are used for high-throughput and (semi-)automatic data analysis. The results are generally classified into four classes (levels) according to the guidelines of the Metabolomics Standards Initiative: Level 1, identification using authentic standard compounds; Level 2, annotation using public databases; Level 3, characterization by deciphering MS/MS data; and Level 4, unknown (not noise) (Sumner et al., 2007). The use of these levels reduces the production of false positive data.

An integrated approach using LC-MS/MS with ^13^C (carbon) labeling and cheminformatics can be used to assign structures to metabolites whose structures have never been recognized before. In most of the general approaches, unknown metabolites remain uncharacterized. The integrative approach is used to identify the elemental compositions of the unknown metabolites based on the numbers of C atoms, determined by comparisons between ^13^C- and non-labeled MS spectra. The MS/MS spectra of the unknown metabolites are then subjected to fragment set enrichment analysis (FSEA) to determine candidates for each metabolite class. This integrative approach was used to characterized 1,133 metabolites, including flavonoids, in 12 angiosperm species (Tsugawa et al., 2019). The results for the characterized flavonoids are summarized in Table 1, along with the flavonoids found in the liverwort *Marchantia polymorpha*, which were analyzed using the same method (Kubo et al., 2018). Our analyses using LC-MS/MS with ^13^C labeling and cheminformatics led to the identification of more flavonoid molecules especially in the group of flavonol and flavone. Prenylated flavonoids have been analyzed by LC-MS/MS less extensively than other flavonoid groups, although a chemical assignment strategy is effective for profiling prenylated flavonoids. It is probably due to the lack of authentic standards and publicly available MS/MS spectra. The exact mass of a known structure or one that is presumed to be prenylated can also be useful for profiling prenylated flavonoids in cases where the ontologies for prenylated flavonoids does not work. This means that the observed MS/MS spectra may not be matched to the library derived from the FSEA.

**Table 1:** Flavonoids experimentally characterized by LC-MS/MS in 12 angiosperm species and *Marchantia polymorpha*.

**Group**	**Representative structure**		**Name**												
			**Marchantiales**	**Asparagales**	**Poales**		**Solanales**			**Gentianales**	**Brassicales**	**Fabales**			
			**Marchantia polymorphia**	**Allium cepa**	**Oryza Sativa**	**Zea mays**	**Lycopersicon esculentum**	**Nicotiana tabacum**	**Solanum tuberosum**	**Optiorriza pumila**	**Arabidopsis thaliana**	**Glycine max**	**Glycyrrhiza glabra**	**Glycyrrhiza uralensis**	**Medicago truncatula**
Flavanone	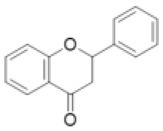	Eriodictyol, hesperetin, isookanin, liquirtigenin, naringenin, sakuranetin, etc.	0 (0)	1 (0)	0 (2)	0 (0)	2 (0)	0 (0)	0 (0)	0 (0)	0 (0)	2 (0)	10 (5)	7 (17)	1 (0)
Flavone	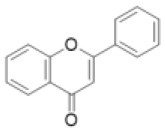	Apigenin, baicalein, chrysoeriol, luteolin, scutellarein, tricin, etc.	5 (3)	1 (5)	30 (19)	23 (17)	2 (0)	2 (13)	3 (0)	1 (0)	0 (0)	10 (4)	15 (2)	11 (5)	12 (0)
Isoflavone	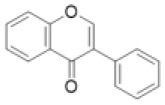	Afrormosin, biochanin, daizein, formononetin, genistein, etc.	0 (0)	0 (0)	0 (0)	1 (0)	0 (0)	0 (6)	0 (0)	8 (0)	0 (0)	18 (18)	14 (8)	17 (3)	5 (0)
Flavanol/flavandiol	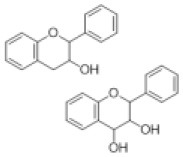	Catechin, procyanidin	0 (0)	0 (0)	0 (0)	0 (0)	0 (0)	0 (0)	0 (0)	0 (0)	0 (0)	0 (0)	2 (0)	2 (0)	0 (0)
Flavonol	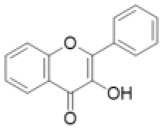	Gossypetin, isorhamnetin, kaempferide, kaempferol, laricitrin, myricetin, patuletin, quercetin, syringetin	0 (0)	10 (3)	2 (0)	6 (3)	7 (0)	3 (1)	10 (3)	0 (0)	9 (1)	6 (9)	19 (2)	19 (0)	15 (0)
Anthocyanidin	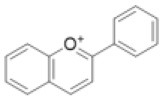	Cyanidin, delphnidin, petunidin, riccionidin, etc.	1 (2)	0 (10)	0 (0)	1 (0)	1 (0)	0 (0)	0 (6)	0 (0)	0 (3)	1 (0)	2 (0)	2 (0)	0 (0)
Prenylated flavonoid	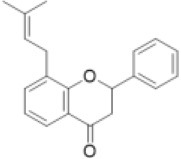	Bolusanthol, gancaonin, glabrol, kievitone, licoisoflavone, sigmoidin, etc.	0 (0)	0 (0)	0 (0)	0 (0)	0 (0)	0 (0)	0 (0)	0 (0)	0 (0)	0 (4)	13 (49)	10 (40)	0 (0)

*The numbers of flavonoids characterized by Tsugawa et al. (2019) are shown. The numbers of flavonoids stored in the Dictionary of Natural Products are shown in parentheses. A conventional type of reverse phase LC-MS/MS was used for these analyses. On the basis of the ontology in the FSEA, the flavonoids are summarized into seven groups. Flavonoids with the prenyl moiety are grouped together as the prenylated flavonoid group. Prenylations (29 patterns) occur in chalcones/dihydrochalcones, flavanones, flavones, flavonols/dihydroflavonols, and isoflavones (Barron and Ibrahim, 1996). Flavonoids consisting of unmodified aglycones and glycosylated, acylated, etherated, and/or prenylated flavonoids were characterized in this study*.

Similarly, recent studies suggest that the tricin metabolism seems to specifically evolve in monocot plants. Tricin serves as a lignin co-monomer and cross couples with monolignols and γ-*p*-coumaroylated monolignols upon cell wall lignification. Recently, tricin was identified as a nucleation site for lignification in grasses (Lan et al., 2015, 2016a; Lam et al., 2017). Poales plants including *Avena sativa, Brachipodium distachyon, Oryza sativa, Triticum durum*, and *Zea mays* accumulate tricin, as does the Fabales plant *Medicago sativa* (Lan et al., 2016b). An integrative approach was used to detect derivatives of apigenin, luteolin, and tricin in *O. sativa, Z. mays*, and *M. truncatula*, and it appears that the total tricin content is much higher than the extractable tricin content in these plants. The integrative approach with an FSEA may be useful for further understanding the role of tricin and its derivatives in the cell wall.

LC-MS/MS-based metabolome analyses have been used to comprehensively analyze the flavonoid molecules in individual plant species. Recent technological developments allow us to perform high-throughput LC-MS/MS analyses. By tracing the ions derived from the aglycones and modified parts of the molecules, or by finding MS/MS similarities, the chemical assignment of flavonoids is easily performed in (semi-)automatic ways with cheminformatic tools across plant species.

Biosynthetic genes for the formation of aglycone or modification can be predicted on the basis of the structure of flavonoids. Functional genomics using both cutting-edge sequencer and more comprehensive and accurate flavonoid profiling allow us to address the evolutionary questions of when and why flavonoids appeared and the biosynthetic pathway are diversified in plants. Furthermore, along with the progress of considerable plant genome projects, it will provide valuable clues to understand the evolutionary traits of flavonoid metabolism in plant kingdom as inconsistencies in the relationships between metabolites and genes that we mentioned in later sections.

## The Flavonoid Biosynthetic Pathways

**Figure 2:**
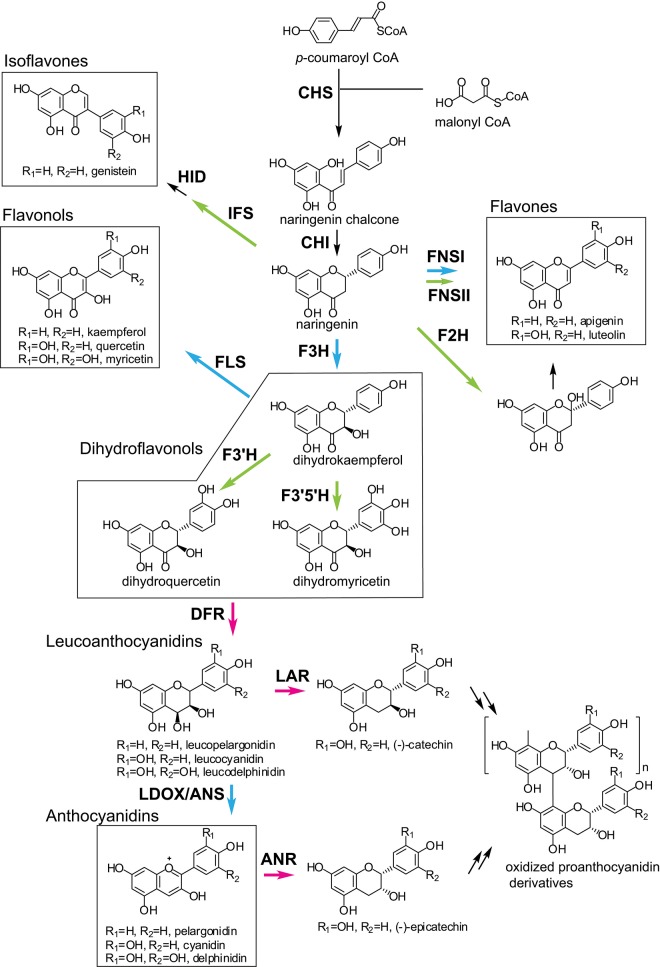
General flavonoid biosynthetic pathways in plants. The arrows in green, blue, and magenta indicate enzymes in the CYP, 2OGD, and SDR superfamilies, respectively. ANR, anthocyanidin reductase; ANS, anthocyanidin synthase; CHI, chalcone isomerase; CHR, chalcone reductase; CHS, chalcone synthase; DFR, dihydroflavonol 4-reductase; F2H, flavanone 2-hydroxylase; F3H, flavanone 3- hydroxylase; F3′H, flavonoid 3′-hydroxylase; F3′5′H, flavonoid 3′5′-hydroxylase; FLS, flavonol synthase; FNS, flavone synthase; HID, 2-hydroxyisoflavanone dehydratase; IFS, Isoflavone synthase; LAR, leucoanthocyanidin reductase; LDOX, leucoanthocyanidin dioxygenase.

The first step in flavonoid biosynthesis is catalyzed by chalcone synthase (CHS) (Figure 2). The substrates *p*-coumaroyl-CoA, derived from the cinnnamate/monolignol (phenylpropanoid) pathway, and malonyl-CoA from the acetate-malonate (polyketide) pathway are converted by CHS to naringenin chalcone. The stereospecific cyclization of naringenin chalcone to naringenin is catalyzed by chalcone isomerase (CHI). This step can also proceed spontaneously.

Naringenin is a general precursor for flavonols, anthocyanins, proanthocyanidins, flavones, and isoflavones. It is converted to dihydrokaempferol by flavanone 3-hydroxylase (F3H) (also referred to as flavanone 3β-hydroxylase). Flavonoid 3′-hydroxylase (F3′H) and flavonoid F3′5′H-hydroxylase (F3′5′H) catalyze the hydroxylation of the C3′ and C3′/C5′ positions of dihydrokaempferol, respectively. Dihydroflavonol 4-reductase (DFR) catalyzes the reduction of the dihydroflavonols to leucoanthocyanidins, which are further converted to anthocyanidins by leucoanthocyanidin dioxygenase/anthocyanidin synthase (LDOX/ANS). The leucoanthocyanidins and anthocyanidins are reduced to flavan 3-ols (e.g., catechin and epicatechin) by leucoanthocyanidin reductase (LAR) and anthocyanidin reductase (ANR), respectively. The dihydroflavonols are also converted to flavonols by flavonol synthase (FLS).

Flavones are synthesized from naringenin by flavone synthase I (FNS I) or flavone synthase II (FNS II) (Martens and Mithofer, 2005). Flavanone 2-hydroxylase (F2H) catalyzes the hydroxylation of flavanones (including naringenin) to 2-hydroxyflavanones, which are subsequently converted to flavones, possibly by an unknown dehydratase (Akashi et al., 1998).

Isoflavone synthase (2-hydroxyisoflavanone synthase, IFS) catalyzes the first step of isoflavone biosynthesis. IFS converts flavanones (e.g., liquirtigenin and naringenin) to 2-hydroxyisoflavanones, then the 2-hydroxyisoflavanones are dehydrated to isoflavones by 2-hydroxyisoflavanone dehydratase (HID) (Akashi et al., 2005).

The flavonoid skeletons mentioned above are highly modified by enzymes such as glycosyltransferases (GTs), acyltransferases, and methyltransferases. Here, we focus on the enzymes involved in the biosynthesis of the flavonoid skeleton molecules.

## Evolutionary History of the Flavonoid Biosynthetic Pathways

Generally, the enzymes in secondary metabolic pathways have been derived from those involved in primary metabolism (Pichersky and Gang, 2000; Moghe and Last, 2015; Carrington et al., 2018), and the enzymes in flavonoid metabolism are no exception. CHS and CHI are derived from enzymes involved in fatty acid metabolism: β-ketoacyl ACP synthase and fatty acid binding protein, respectively (Ngaki et al., 2012; Weng and Noel, 2012a). CHS and β-ketoacyl ACP synthase are members of the type III polyketide synthase (PKS) family (Schuz et al., 1983). The enzymes in later steps of the flavonoid biosynthetic pathways belong to families such as the 2OGD, CYP, and short-chain dehydrogenase/reductase (SDR) superfamilies (Table 2). The members of these superfamilies are widely involved in primary and secondary metabolisms, suggesting that plants have acquired the enzyme functions in later biosynthetic pathways via gene duplication and evolution of new functions for the duplicated gene products.

**Table 2:** The origin or gene family of flavonoid biosynthetic genes.

**Gene**	**Abbreviation**	**Origin/gene family**
*Chalcone synthase*	CHS	Type III polyketide synthase
*Chalcone isomerase*	CHI	Fatty acid binding protein
*Chalcone isomerase-like*	CHIL	Fatty acid binding protein
*Flavanone 3-hydroxylase*	F3H	2-oxoglutarate-dependent dioxygenase (DOXC28)
*Flavone synthase I*	FNS I	2-oxoglutarate-dependent dioxygenase (DOXC28)
*Flavone synthase II*	FNS II	Cytochrome P450 (CYP93B)
*Isoflavone synthase*	IFS	Cytochrome P450 (CYP93C)
*Flavonoid 3′-hydroxylase*	F3′H	Cytochrome P450 (CYP75)
*Flavonoid 3′,5′-hydroxylase*	F3′5′H	Cytochrome P450 (CYP75)
*Flavonol synthase*	FLS	2-oxoglutarate-dependent dioxygenase (DOXC47)
*Dihydroflavonol 4-reductase*	DFR	Short-chain dehydrogenase (SDR108E)
*Leucoanthocyanidin dioxygenase*/*anthocyanidin synthase*	LDOX/ANS	2-oxoglutarate-dependent dioxygenase (DOXC47)
*Anthocyanidin reductase*	ANR	Short-chain dehydrogenase (SDR108E)
*Leucoanthocyanidin reductase*	LAR	Short-chain dehydrogenase (SDR460A)
*Glycosyltransferase*	GT	Family 1 glycosyltransferase, glycoside hydrolase family 1
*Acyltransferase*	AT	BAHD acyltransferase, serine carboxypeptidase-like acyltransferase
*Methyltransferase*	MT	Methyltransferase

Based on the distribution patterns of the flavonoid subclasses, it has been suggested that the flavonoid biosynthetic pathways may have evolved via a series of steps, and that the first flavonoid biosynthetic enzymes were CHS, CHI, and F3H (Stafford, 1991; Rausher, 2006). It has also been proposed that CHS evolved first, followed by F3H and then CHI, because CHS catalyzes the first committed step in the pathways, and the step catalyzed by CHI can also proceed spontaneously (Rausher, 2006). Flavonoids are widely distributed among mosses, liverworts, and vascular plants, but are not found in hornworts. Algae generally contain no flavonoids (Rausher, 2006), but they have been found in a few evolutionary divergent lineages of microalgae (Goiris et al., 2014). These observations suggest that the ability to produce flavonoids may have evolved multiple times, or that the ability was widely lost during evolutionary processes. An analysis of the evolutionary rates of six genes involved in anthocyanin biosynthesis indicated that the upstream genes (*CHS, CHI*, and *F3H*) evolved more slowly than the downstream genes (*DFR, LDOX/ANS*, and *UDP-glucose:flavonoid 3-O-glucosyltransferase*) (Rausher et al., 1999). The upstream genes may be evolutionarily constrained due to their profound effects on the pathways. In addition, the genes encoding transcription factors that regulate anthocyanin biosynthesis have evolved more rapidly than the structural genes (Rausher et al., 1999).

## The Evolutionary History of Flavonoid Biosynthetic Genes

### CHS Is a Representative of the Type III PKS Superfamily

**Figure 3:**
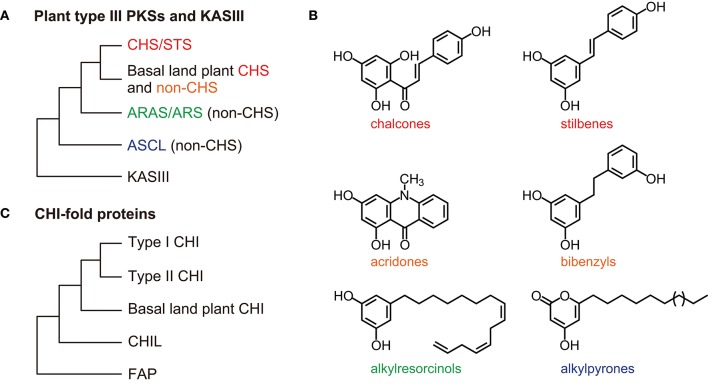
General overview of the type III PKS and CHI-fold protein phylogenies. **(A)** Relationships among the CHS/STS proteins, the basal land plant CHS, and non-CHS proteins of the plant type III PKSs. The non-CHS proteins include the ARAS/ARS proteins and the ASCL families. The overall three-dimensional protein structure is conserved in the type III PKSs and an *E. coli* KASIII enzyme (the αβαβα-fold). **(B)** Examples of type III PKS products. **(C)** The CHI-fold proteins in the CHI, CHIL, and FAP families share a common folded protein structure (the open-faced β-sandwich fold). ARAS, alkylresorcylic acid synthase; ARS, alkylresorcinol synthase; ASCL, anther-specific chalcone synthase-like enzyme; CHIL, CHI-like protein; CHI, chalcone isomerase; CHS, chalcone synthase; FAP, fatty-acid-binding protein; KASIII, 3-ketoacyl-ACP synthase isoform III enzyme; PKSs, polyketide synthases; STS, stilbene synthase.

The *CHS* genes are widely distributed in plants, from bryophytes to angiosperms (Jiang et al., 2008; Shimizu et al., 2017; Liou et al., 2018), but they have not been found in other organisms. Every land plant species with available genomic data has at least one putative *CHS* gene (Shimizu et al., 2017). CHS is a member of the type III PKS superfamily, which provides diverse polyketide scaffolds of secondary metabolites (Figure 3) (Winkel-Shirley, 2001; Austin and Noel, 2003; Abe and Morita, 2010). The type III PKSs belong to the thiolase superfamily (Jiang et al., 2008). They are found in land plants, microalgae, fungi, and bacteria, but are not found in animals or archaea (Shimizu et al., 2017). The plant type III PKSs retain the overall folded protein structure and the Cys-His-Asn catalytic triad that characterize the *Escherichia coli* 3-ketoacyl-ACP synthase isoform III (KASIII) enzyme, which is involved in *de novo* fatty acid synthesis (Ferrer et al., 1999; Austin and Noel, 2003). The number of land plant type III *PKS* genes is highly variable among species in the same taxa; for instance, eudicot species have two to 42 genes, whereas the numbers in fungi and bacteria are relatively low (less than five) (Shimizu et al., 2017). The abundance of type III PKSs may contribute the variety of specialized plant metabolites.

The functional diversity of the type III PKSs is generally derived from differences in the starter molecules, the numbers of chain elongation steps, and the mechanisms of the cyclization reactions (Austin and Noel, 2003; Abe and Morita, 2010). The stilbene synthases (STSs), which produce stilbenes such as resveratrol, are also type III PKSs. CHSs and STSs generate the same intermediates from the same starter molecules using the same chain elongation steps, but they catalyze different intra-molecular cyclizations and produce different products (Austin et al., 2004). The CHSs and STSs from the same plant genera are usually classified as the closest neighbors in phylogenetic trees (Figure 3). Data from angiosperms (*Vitis vinifera, Arachis hypogaea*, and *Sorghum bicolor*), gymnosperms (*P. sylvestris*), and ferns (*Psilotum nudum*) suggest that after diverging, the STSs and CHSs evolved independently (Yu et al., 2005; Weng and Noel, 2012b).

### Basal Land Plant CHSs and Non-CHSs

The eudicot *Arabidopsis thaliana* contains four type III *PKS* genes, including a single functional *CHS* (Kim et al., 2010). In contrast, the bryophyte moss *Physcomitrella patens* has around 19 *CHS* family genes and four other type III *PKS* genes (Jiang et al., 2006; Wolf et al., 2010). Five of the moss *CHS* family genes were derived from a whole genome duplication, and four are suggested to be derived from segmental duplication and transposition (Wolf et al., 2010). Four of the genes are upregulated by broad-band UV-B irradiation, and the moss plants show increased levels of a flavonol derivative under UV-B illumination. These observations support the hypothesis that the genes and enzymes involved in the UV stress response evolved with the water-to-land transition, when early plants were exposed to increased levels of sunlight (Wolf et al., 2010).

In the bryophyte liverwort *M. polymorpha*, UV-B irradiation and nutrient deprivation significantly increase the total flavone glycoside content (Albert et al., 2018; Clayton et al., 2018). *M. polymorpha* has 24 *CHS* family genes (Bowman et al., 2017) and one of these is significantly upregulated by UV-B stress treatment. This induction was enhanced in transgenic *Marchantia* plants that overexpressed the gene encoding the UV RESISTANCE LOCUS8 (UVR8) photoreceptor (Clayton et al., 2018). Furthermore, transgenic *Marchantia* plants overexpressing a *MpMyb14* transcription factor gene showed increased expression levels of the same *CHS* gene under normal growth conditions (Albert et al., 2018). Knockout *Mpmyb14* mutants were partially impaired by an increase in *CHS* expression levels under nitrogen deficient conditions (Kubo et al., 2018). The results suggest that these liverwort species have at least one *CHS* gene that is activated by the UVR8 signal transduction pathway.

Phylogenetic analyses show close relationships between basal land plant (bryophyte and lycophyte) CHSs and non-CHS type III PKSs (Figure 3) (Wanibuchi et al., 2007; Yu et al., 2018). The non-CHS group includes many enzymes involved in the biosynthesis of secondary metabolites, such as acridone synthases, pyrone synthases, bibenzyl synthases, and *p*-coumaroyltriacetic acid synthases (Winkel-Shirley, 2001). These non-CHS PKS enzymes evolved through repeated gene duplication, mutation, and functional diversification from their ancestral plant enzymes.

Ectopic expression of a *CHS* gene from either the bryophyte *P. patens* or the lycophyte *Selaginella moellendorffii* can partially complement the phenotype of an *A. thaliana CHS*-null mutant, *transparent testa 4* (Liou et al., 2018). Crystal structures of CHSs from *P. patens, S. moellendorffii*, the monilophyte *Equisetum arvense*, the gymnosperm *Pinus sylvestris*, and the angiosperm *A. thaliana* revealed that the reactivity of the catalytic Cys residue (Cys164 in *M. sativa* CHS2) has changed during the 500 million years of evolution of land plants. The Cys residues in the three recent lineages (monilophytes, gymnosperms, and angiosperms) are present in the thiolate anion form, which gives them stronger nucleophilic power (Liou et al., 2018).

The type III PKSs show broad substrate promiscuity. CHSs do not accept bulky substrates, but the lycophyte *Huperzia serrata* HsPKS1 exhibits remarkable substrate tolerance and catalytic potential (Wanibuchi et al., 2007; Morita et al., 2011). *In vitro*, HsPKS1 produces naringenin chalcone and other polyketides, including aromatic tricyclic pyridoisoindole compounds, which are not found in natural products. A single amino acid replacement in HsPKS1 increases its active-site cavity volume and alters the product chain length and the mechanism of the cyclization reaction. This substrate promiscuity in the type III PKSs provides diverse polyketide scaffolds for the subsequent biosynthesis of secondary metabolites in land plants.

### The ARAS/ARS and ASCL Families

Plant non-CHS type III PKSs also synthesize polyketides from fatty acyl-CoA substrates. Phenolic lipids such as alkylresorcinols are synthesized by alkylresorcinol synthases (ARSs) and alkylresorcylic acid synthases (ARASs) in the monocots *S. bicolor* and *O. sativa*, respectively (Cook et al., 2010; Matsuzawa et al., 2010). Alkylresorcinols in grain crop species show anti-fungal and allelopathic activities.

Hydroxyalkyl-α-pyrone compounds, which are precursors of sporopollenin in the pollen wall exine, are synthesized by anther-specific chalcone synthase-like enzymes (ASCLs) that are specifically and transiently expressed in *A. thaliana* anthers (PKSA/LAP6 and LAP5/PKSB) (Dobritsa et al., 2010; Kim et al., 2010). The *pksa pksb* double mutant plants are male sterile.

Phylogenetic analyses show that the ARAS/ARS and ASCL families are classified into distinct groups (Figure 3) (Shimizu et al., 2017). Bacteria, fungi, and mosses also have genes encoding type III PKSs that produce these long-chain fatty acyl containing-polyketides (Colpitts et al., 2011; Shimizu et al., 2017; Li et al., 2018). Therefore, these type III PKSs are involved in lipid metabolism across three kingdoms of living organisms.

### CHI-Fold Proteins: CHIs and CHILs

The CHIs and CHI-like proteins (CHILs) are members of the CHI-fold family, which also includes the fatty-acid-binding proteins (FAPs) that are involved in fatty acid biosynthesis (Figure 3). These proteins share a common folded three-dimensional structure (Jez et al., 2000; Ngaki et al., 2012; Kaltenbach et al., 2018). The FAP family is distributed in many bacteria, fungi, and plant species. CHILs partly lack the catalytic amino acid residues conserved in CHIs, but they bind with CHSs and enhance their activity (Ban et al., 2018). Phylogenetic and genomic analyses of CHI-fold proteins suggest that CHILs first appeared in mosses and evolved from FAPs, and then served as the ancestors of CHIs (Ngaki et al., 2012; Morita et al., 2014; Jiang et al., 2015). The CHILs form a group that is distinct from the CHI and FAP groups (Figure 3). The moss *P. patens* has two *CHIL* and four *FAP* genes, but does not appear to have any *CHI* genes (Ngaki et al., 2012; Cheng et al., 2018). The liverwort *M. paleacea* has a *CHI*, a *CHIL*, and two *FAP* genes (Cheng et al., 2018) and the lycophyte *S. moellendorffii* has a *CHI*, a *CHIL*, and three *FAP* genes. *A. thaliana* has a *CHI*, a *CHIL*, and three *FAP* genes whereas the legume *Glycine max* has a type I *CHI*, three type II *CHIs*, two *CHILs*, and six *FAP* genes (Dastmalchi and Dhaubhadel, 2015; Ban et al., 2018). These results suggest that the number of *CHI* and *CHIL* genes remains low in many plant lineages, but that leguminous plants have several *CHI* genes.

### Type I and Type II CHIs in Vascular Plants

There are two types of CHI. Type I CHIs are ubiquitous in vascular plants, whereas type II CHIs are specific to legumes and are involved in flavonoid synthesis during nitrogen-fixing root nodule symbioses (Shimada et al., 2003; Subramanian et al., 2007). Phylogenetic analyses show that the type II CHIs form a distinct group from the type I CHIs (Figure 3). The type II CHIs likely evolved from ancestral CHI-fold proteins (Cheng et al., 2018).

The type II CHIs isomerize both naringenin chalcone and isoliquiritigenin (6′-deoxychalcone) to produce (2*S*)-naringenin and liquiritigenin (5-deoxyflavanone), respectively. The model legume *Lotus japonicas* has a type I and three type II *CHI* genes (Shimada et al., 2003). These four *CHI* genes form a tandem cluster within a 15-kb region of the genome. In soybean, a type I and two type II *CHI* genes are organized in a gene cluster on chromosome 20 (Dastmalchi and Dhaubhadel, 2015), and are probably derived from tandem gene duplications. The role of the CHIs in legume symbiosis remains unclear. Nitrogen-fixing root nodule symbioses are found in four angiosperm orders: Fabales, Fagales, Cucurbitales, and Rosales (the latter three are known as actinorhizal plants). The expression of a *CHI* gene is upregulated in nodules of the actinorhizal plant *Datisca glomerata* (Gifford et al., 2018), suggesting that these CHIs contribute to increases in flavonoid contents.

### CHIs in Basal Land Plants

The *A. thaliana* chi*-*deficient *tt5* mutants produce pale yellow seeds due to significant reductions in proanthocyanidin production. This phenotype is largely complemented by ectopic expression of a *CHI* gene from either the liverwort *M. paleacea* (*MpCHI1*) or the lycophyte *S. moellendorffii* (*SmCHI1*) (Cheng et al., 2018). The CHIs from basal land plants have broad substrate specificities and are more like the type II CHIs than the type I CHIs. Phylogenetic analyses show that MpCHI1 and SmCHI1 form separate groups from the type I and type II CHIs (Figure 3) (Cheng et al., 2018; Kaltenbach et al., 2018).

The liverwort *M. polymorpha* also has a single *CHI* gene (Clayton et al., 2018), and *Marchantia chi* mutants do not contain detectable levels of flavone compounds. These *chi* mutants are highly sensitive to UV-B stress treatment. These results indicate that this basal plant species already has a gene encoding a *bona fide* CHI to catalyze the cyclization of naringenin chalcone in the flavonoid biosynthetic pathway.

### The CHIL Family

CHILs are categorized as type IV CHI-fold proteins and are found in basal and higher plant species including mosses, liverworts, lycophytes, ferns, gymnosperms, and angiosperms. CHILs do not have *bona fide* CHI activity. However, the RNAi knockdown of *CHIL* expression in *Petunia hybrida* and *Torenia hybrida* resulted in decreased levels of total flavonoids in the flowers (Morita et al., 2014). Three independent *Ipomoea nil* (Japanese morning glory) mutants with alterations in *enhancer of flavonoid production* (a *CHIL* gene) showed pale-colored flower phenotypes (Morita et al., 2014). *CHIL* loss-of-function mutants in *A. thaliana* show reductions in the levels of proanthocyanidin and flavonols in seeds, and flavonols in leaves (Jiang et al., 2015). However, the *A. thaliana CHIL* gene could not rescue the phenotypes of the *tt5* mutants (Jiang et al., 2015). The liverwort *M. polymorpha* has a *CHIL* gene (Clayton et al., 2018), and the total flavone content is reduced in *Marchantia chil* mutants under normal growth conditions and under UV-B treatment. Thus, the CHILs enhance total flavonoid production but have roles that are distinct from those of the CHIs. As mentioned above, it was recently shown that CHILs from various plant lineages (*A. thaliana, O. sativa, S. moellendorffii*, and *P. patens*) can bind CHSs and boost CHS activity (Ban et al., 2018). CHIs also bind CHSs (Jorgensen et al., 2005), and it may be that the type I and type II CHIs evolved from CHILs and gained their CHI activity during subsequent evolutionary processes (Ban et al., 2018).

### Acquisition of Enzymatic CHI Activity During Evolution

The emergence of enzymatic CHIs in plants is an interesting topic in protein evolution, because the CHILs and FAPs are non-enzymatic proteins. The cleft in the CHI active site consists of three highly conserved amino acid residues (Arg36, Thr48, and Tyr106 in the *M. sativa* type II CHI sequence) and their neighboring residues are also conserved (Jez et al., 2000; Ngaki et al., 2012; Kaltenbach et al., 2018). The common ancestral proteins of the CHIs and CHILs were inferred in an extensive phylogenic analysis, and it appears that all three key catalytic residues were conserved in the ancestral proteins but were inactive (Kaltenbach et al., 2018). The authors performed a stepwise, activity-based screening of recombinant ancestral proteins using an *E. coli* expression system. The results indicated that mutations in amino acid residues other than the catalytic residues were required to initiate CHI evolution, and to acquire CHI catalytic activity.

### The 2-Oxoglutarate-Dependent Dioxygenase Family: F3H, FNS I, FLS, and LDOX/ANS

The 2OGD superfamily is one of the largest protein families in the plant kingdom. Its members are widely distributed in bacteria, fungi, plants, vertebrates, and even viruses (van den Born et al., 2008; Farrow and Facchini, 2014; Markolovic et al., 2015; Wu et al., 2016). The 2OGDs are non-heme iron containing enzymes that are localized in the cytosol. 2OGDs incorporate 2-oxoglutarate (2OG or α-ketoglutarate) and activated O_2_ into a variety of substrates to form the oxidized products along with succinate and CO_2_ (RH + 2OG + O_2_ → ROH + succinate + CO_2_). 2OGDs catalyze various oxidative reactions including hydroxylation, halogenation, desaturation, and epimerization (Martinez and Hausinger, 2015) and play important roles in DNA and RNA repair, fatty acid metabolism, oxygen sensing, and biosynthesis of natural products (Farrow and Facchini, 2014; Hagel and Facchini, 2018; Herr and Hausinger, 2018; Islam et al., 2018).

In plants, 2OGDs are involved in histone demethylation, iron sensing, phytohormone metabolism, and the biosynthesis of secondary metabolites (reviewed in Farrow and Facchini, 2014). A phylogenetic analysis of plant 2OGDs from *Chlamydomonas reinhardtii, P. patens, S. moellendorffi, Picea abies, O. sativa*, and *A. thaliana* found 3 classes, which the authors named DOXA, DOXB, and DOXC (Kawai et al., 2014). Each class contains proteins from all six species. The DOXA class contains homologs of *E. coli* AlkB; these enzymes are involved in DNA repair (Lindahl et al., 1988; Meza et al., 2012; Mielecki et al., 2012). The DOXB class contains prolyl 4-hydroxylases that catalyze the hydroxylation of proline residues in plant cell wall proteins (Hieta and Myllyharju, 2002). Proteins in the DOXC class are involved in phytohormone metabolism and the biosynthesis of secondary metabolites including flavonoids, terpenoids, alkaloids, and glucosinolates. The numbers of genes encoding DOXA and DOXB enzymes are limited in the six species, however, the DOXC genes are significantly expanded in the land plants (Kawai et al., 2014).

Four flavonoid biosynthetic enzymes, F3H, FNS I, FLS, and LDOX/ANS belong to the DOXC class. A phylogenetic analysis showed that the genes in the DOXC class can be classified into over 50 clades (Kawai et al., 2014). The *F3H* and *FNS I* genes are in the DOXC28 clade while the *FLS* and *LDOX/ANS* genes are in DOXC47. Among the flavonoid biosynthetic genes in the *2OGD* superfamily, it has been proposed that *F3H* was the first to appear (Rausher, 2006). FNS I seems to exist only in the Apiaceae, and it is likely that *FNS I* evolved from *F3H* as a paraphyletic gene (Martens et al., 2003; Gebhardt et al., 2005).

*Arabidopsis thaliana* contains one *F3H* gene, six *FLS* genes (*AtFLS1*–*AtFLS6*), one *LDOX/ANS* gene, and no FNS gene. AtFLS1 is the major FLS (Owens et al., 2008a; Saito et al., 2013). An analysis of structural divergence between duplicated genes showed that transposed duplication (<16 million years ago) explains the relationship between AtFLS6 (At5g43935) and AtF3H (At3g51240) (Wang et al., 2013). Furthermore, the relationship between AtFLS1 (At5g08640) and AtFLS5 (At5g63600) is likely explained by a whole genome duplication, and those between AtFLS2 (At5g63580) and AtFLS3 (At5g63590), AtFLS3 and AtFLS4 (At5g6359580), and AtFLS4 and AtFLS5 are most likely explained by tandem local duplications (Wang et al., 2013). The *A. thaliana* AtLDOX/ANS has been shown to produce flavonols *in planta* (Preuss et al., 2009). Furthermore, FLS and LDOX/ANS can partially complement F3H function *in vivo*, and this results in the leaky phenotype of *tt6* mutants with null mutations in *AtF3H* (Owens et al., 2008b).

Together, the results suggest that *F3H* is the ancestral *2OGD* gene for flavonoid biosynthesis, and that *FLS* and *FNS I* evolved via divergence from *F3H*. No apparent orthologs of either the DOXC28 or DOXC47 clade genes were found in *P. patens* and *S. moellendorffi*, even though these plants produce flavonols (*P. patens*) and flavones (*S. moellendorffi*) (Wolf et al., 2010; Weng and Noel, 2013). However, the liverwort *Plagiochasma appendiculatum* has an active FNS I (PaFNS I), and a phylogenic analysis revealed that PaFNS 1 is related to the angiosperm FNS I and F3H proteins, even though it is not in the same clade as them (Han et al., 2014). These data suggest that in *Physcomitrella* and *Selaginella*, the 2OGDs are present in distinct clade(s), or that unrelated enzymes perform the same functions as F3H, FNS, and/or FLS.

The PaFNS I can convert naringenin to either 2-hydroxynaringenin or apigenin (Han et al., 2014). The common horsetail *E. arvense* L also has 2OGD-type FNS I activity (Bredebach et al., 2011). Further research in the *2OGD* genes of bryophytes, lycophytes, and ferns will help to clarify their evolutionary processes.

### The Cytochrome P450 Superfamily: FNSII, F2H, IFS, F3′H, and F3′5′H

The CYPs are widely distributed in viruses, archeae, bacteria, and eukaryotes. They catalyze monooxygenase/hydroxylation reactions in various primary and secondary metabolic processes by insertion of an O atom from molecular O_2_ (Mizutani and Ohta, 2010). In eukaryotes, the CYPs are heme-containing membrane proteins localized on the cytosolic surface of the endoplasmic reticulum.

In plants, the CYPs form the largest superfamily of enzymes and account for about 1% of the total number of gene products (Mizutani and Ohta, 2010; Nelson and Werck-Reichhart, 2011; Kawai et al., 2014) The CYPs are categorized into families (e.g., CYP75) that have ~40% or more amino acid sequence identity, and those with 55 % or more identity are categorized into subfamilies (e.g., CYP75A). Furthermore, the plant CYP families (CYP71 to CYP99 and CYP701–) can be classified into clans whose members are derived from single ancestors. The land plant CYPs form 11 clans and seven of these (clans 51, 74, 97, 710, 711, 727, and 746) consist of single CYP families, while the remaining four (clans 71, 72, 85, and 86) include proteins in multiple CYP families (Nelson and Werck-Reichhart, 2011). Green algae contain CYPs in five single family clans (clans 51, 97, 710, 711, and 746), and members of these clans are involved in fundamental biological processes such as biosynthesis of sterols, xanthophylls, and phytohormones (Nelson, 2006; Nelson and Werck-Reichhart, 2011). Therefore, it is likely that these clans include the ancestral CYPs. The multi-family CYP clans have become highly diversified during plant evolution (Nelson and Werck-Reichhart, 2011). Some CYP families in these clans (clans 71, 72, 85, and 86) are present in bryophytes and/or liverworts but not in green algae. Additional novel CYP families were gained in stepwise processes following the evolution of vascular plants.

Flavonoid biosynthetic enzymes (members of the CYP75 and CYP93 families) are classified into clan 71. This clan contains the largest number of CYP families, and in addition to the flavonoid biosynthetic enzymes, it includes families involved in the biosynthesis of phenylpropanoids (CYP73, CYP84, CYP98), alkaloids (CYP80, CYP82, CYP719), terpenoids (CYP76, CYP99, CYP705, CYP706, CYP726), and glucosinolates (CYP79, CYP83). The clan 71 families CYP73, CYP74, CYP78, CYP88, CYP98, CYP701, CYP703, CYP736, and CYP761 are present in bryophytes, suggesting that these may be ancestral families.

### The CYP75 Family: F3′H and F3′5′H

The F3′H and F3′5′H enzymes generally belong to the CYP75B and CYP75A families, respectively. The CYP75 family enzymes from monocots and dicots form distinct clusters within each subfamily, suggesting that the F3′H and F3′5′H functions were established before the divergence of monocots and dicots. However, there are some exceptions. In the Asteraceae, F3′5′H belongs to the CYP75B subfamily and forms a distinct cluster from F3′H (Seitz et al., 2006). This suggests that the Asteraceae F3′H gained F3′5′H activity before speciation but after the separation of the monocots and dicots. Similarly, the rice CYP75B4 catalyzes the 5′-hydroxylation of 3′-methoxyflavone chrysoeriol, and also functions as an F3′H (Lam et al., 2015). Rice contains CYP75B3 as an F3′H and CYP75A11 as a non-functional F3′5′H. Such CYP gene distributions in the CYP75A and CYP75B subfamilies are also found in other Poaceae plants. These data suggest that the CYP75A and CYP75B subfamilies separated before divergence of monocots and dicots, and that genes in the CYP75B subfamily later gained F3′5′H activity, at least in the Asteraceae and Poaceae.

*Arabidopsis thaliana* has a single gene for F3′H (CYP75B1, At5g07990) and no genes corresponding to F3′5′H, FNS II, or F2H. The CYP75B1 gene appears to be related to the CYP701A (At5g25900) gene, which encodes an enzyme involved in gibberellin biosynthesis, via a transposed duplication that occurred 16–107 million years ago and it was proposed that CYP75B1 is the parental locus of CYP701A (Wang et al., 2013). However, the CYP701 family is distributed among bryophytes and vascular plants while the CYP75 family is found in gymnosperms and angiosperms. In addition, a phylogenetic analysis suggested that the moss/liverwort-specific CYP761 family is closely related to the CYP75 family (Nelson and Werck-Reichhart, 2011). These data suggest that either CYP701 or CYP 761 is the ancestral family of CYP75. Although 3′-hydroxylated flavone derivatives were detected in *M. polymorpha* and *S. moellendorffii* (Markham et al., 1998), CYP75 family members can be found only in gymnosperms and angiosperms (Nelson and Werck-Reichhart, 2011). Therefore, in bryophytes, lycophytes, and ferns, enzymes from other CYP families may function as F3′H and/or F3′5′H.

Such inconsistencies in the relationships between metabolites and genes are also observed in phenylpropanoid metabolism. Among the CYPs involved in phenylpropanoid metabolism from clan 71, CYP73A (cinnamate 4-hydroxylase, C4H), CYP98A (*p*-coumaroyl shikimate 3′-hydroxylase, C3′H), CYP73, and CYP98 first appeared in liverworts and mosses, while CYP84 (ferulate 5-hydroxylase, F5H) is found only in angiosperms (Nelson and Werck-Reichhart, 2011). However, the syringyl lignin units from the phenylpropanoid pathway are distributed in land plants (Bowman et al., 2017), suggesting that genes in other CYP families may have F5H activities. In fact, the *Selaginella* CYP788A1 functions as a F5H (Weng et al., 2008).

### The CYP93 Family: FNS II, F2H, and IFS

A genome-wide analysis of the CYP93 family genes from 60 green plants indicated that the CYP93 family is found only in angiosperms (Du et al., 2016). Among the 10 subfamilies (CYP93A–CYP93K), CYP93A is the ancestral group distributed in both monocots and dicots; CYP93B and CYP93C are distributed only in dicots; CYP93G and CYP93J are found only in monocots; and CYP93E and CYP93F are specific to legumes and grasses, respectively. Thus, the CYP93 family shows plant lineage-specific evolution.

The CYP93 family contains enzymes involved in the biosynthesis of flavones and isoflavones (FNS II, F2H, and IFS). The monocot FNS II and F2H belong to the CYP93G subfamily, while those in the dicots are categorized in the CYP93B subfamily. Therefore, phylogenetic analyses suggest that the functions of the FNSII and F2H enzymes were established after the divergence of monocots and dicots. The IFS enzyme in legumes is a member of the CYP93C subfamily, which may be derived from the CYP93B subfamily (Du et al., 2016).

As with the CYP75 family, there are inconsistencies in the relationships between metabolites and genes in the CYP93 family. The CYP93 family genes are found only in angiosperms (Nelson and Werck-Reichhart, 2011), but flavones are widely distributed in plants, from bryophytes to angiosperms (Figure 1). This suggests that enzymes involved in flavone biosynthesis belong to the CYP93 family in some species of angiosperms, but that different CYP families and/or other enzymes play the same roles in other plant taxa. For example, the Apiaceae family adapted the 2OGD-type FNS I rather than the CYP-type FNS II to produce flavones (Gebhardt et al., 2007).

Isoflavonoids are the typical flavonoids found in legumes, and isoflavone biosynthetic genes are found only in legumes. However, isoflavonoids are also found in non-legume plants including *Iris*, which contains a wide variety of isoflavones, some mosses (e.g., *Bryum capillare*), gymnosperms, and monocot and dicot angiosperms (Dewick, 1994; Lapcik, 2007). This suggests that plants independently acquired the ability to produce isoflavonoids during the evolution of the CYP families, except for CYP93C and/or other non-CYP enzymes that function as IFSs.

### The Short-Chain Dehydrogenase/Reductase Family: DFR, ANR, and LAR

*DFR, ANR*, and *LAR* are members of the SDR superfamily, which is widely distributed in viruses, archaea, prokaryotes, and eukaryotes (Jornvall et al., 1999; Kavanagh et al., 2008). The SDRs constitute one of the largest NAD(P)(H) dependent oxidoreductase families and are involved in the primary metabolism of lipids, carbohydrates, and hormones, and the secondary metabolism of molecules such as terpenoids, alkaloids, and phenolic compounds (Jornvall et al., 1999; Kavanagh et al., 2008; Tonfack et al., 2011). In spite of the low overall sequence similarities among SDRs (15–30 %), the SDRs possess a conserved 3D structure consisting of a Rossmann-fold β-sheet surrounded by α-helices for nucleotide binding. Generally, the SDRs can be classified into several types (“classical,” “extended,” “intermediate,” “divergent,” and “complex”) based on their primary structures, cofactor binding motifs, and active sites (Kavanagh et al., 2008; Moummou et al., 2012). A recent study revealed that the “intermediate” and “complex” types are not found in plants while the types “atypical” and “unknown” are found in plants. Thus, the plant SDRs can be categorized into five types: “classical,” “divergent,” “extended,” “atypical,” and “unknown”) (Moummou et al., 2012). Among the plant SDRs, “classical” and “extended” are the major types, as they are in other organisms. The “classical” type is composed of about 250 amino acid residues, and the “extended” type has a domain of 100 additional amino acid residues in the C-terminal region. The “atypical” type, an uncommon type of SDRs, was included in the SDR family because of its Rossmann-fold structure, which is typical of SDRs (Moummou et al., 2012).

A genome sequence analysis using 10 species including *C. reinhardtii, P. patens, S. moellendorffii*, four dicots (*A. thaliana, Populus trichocarpa, V. vinifera*, and *G. max*) and three monocots (*O. sativa, S. bicolor*, and *Z. mays*) showed that most plant SDRs can be classified into 49 families that are distributed among the five types mentioned above (Moummou et al., 2012). The DFR and ANR enzymes are classified into the SDR108E family in the “extended” type. The LAR enzymes belong to the SDR460A family, which is an “atypical” type (Kallberg et al., 2010; Moummou et al., 2012).

### The SDR108E Family: DFR and ANR

Compared to other families that contain a few genes per species, the SDR108E family contains the largest number of SDR genes; for example, this family includes 24 genes in *A. thaliana* and 44 genes in *O. sativa*. Furthermore, the SDR108E family shows the lowest average sequence identity, indicating that the SDR108E family genes are highly diversified (Moummou et al., 2012). The distribution of SDR108E family genes in 10 species indicates that this family has expanded significantly in vascular plants.

In addition to the DFR and ANR enzymes, the SDR108E family contains other enzymes involved in secondary metabolism, including cinnamoyl-CoA reductase (CCR) for lignin biosynthesis, and phenylacetaldehyde reductase for the production of volatile 2-phenylethanol. Furthermore, enzymes involved in phytohormone metabolism, such as phaseic acid reductase for abscisic acid catabolism (Weng et al., 2016) and BEN1 for brassinosteroid homeostasis (Yuan et al., 2007) belong to this family. Each enzyme type forms a distinct cluster in the phylogenetic tree. The CCR and phenylacetaldehyde reductase clusters contain enzymes from *P. patens* and *S. moellendorffii*, whereas the clusters of DFRs, ANRs, phaseic acid reductases, and BEN1 enzymes are derived from flowering plants. These data suggest that these DFRs, ANRs, and other enzymes appeared more recently than the CCRs and phenylacetaldehyde reductases.

Other SDR families show similar gene expansion patterns to that of SDR108E. These families (SDR110C, SDR114C, SDR65C, and SDR460A) contain genes involved in the biosynthesis of alkaloids, terpenoids, phenylpropanoids, and phytohormones (Moummou et al., 2012). SDR families that are less diversified contain genes involved in primary metabolism, such as lipid and chlorophyll biosynthesis (Moummou et al., 2012).

### The SDR460A Family: LAR

The SDR460A family is also referred to as the PIP family, named after the first three enzymes (pinoresinol-laricirecinol reductase, isoflavone reductase, and phenylcoumaran benzylic ether reductase) that were discovered to belong to this family (Gang et al., 1999; Min et al., 2003; Wang et al., 2006). In addition, vestitone reductase, eugenol synthase, and isoeugenol synthase also belong to this family (Koeduka et al., 2008). Pinoresinol-laricirecinol reductase and phenylcoumaran benzylic ether reductase function in the lignan biosynthetic pathway; isoflavone reductase and vestitone reductase are involved in isoflavonoid biosynthesis; and eugenol synthase and isoeugenol synthase are involved in the biosynthesis of volatile phenylpropenes. Thus, the SDR460A family members are involved in the biosynthesis of various phenolic compounds. The SDR460A family members (“atypical” type SDRs) are limited to and has greatly expanded in vascular plants (Moummou et al., 2012), suggesting that they are needed for vascular plant prosperity.

A phylogenetic analysis of LARs from various plants revealed that the plant LARs can be classified into two clusters: proteins from dicotyledons, and proteins from monocotyledons and gymnosperms (Wang et al., 2018). Therefore, the monocotyledon LARs are more closely related to the gymnosperm LARS than the dicotyledon LARS.

## Future Perspectives

The progress in metabolomics technologies including chemoinformatics and the abundant genomic information of flavonoid biosynthetic genes facilitated a fully understanding of evolution of the flavonoid/phenylpropanoid metabolisms in plant kingdom. In this review, we have focused on the evolution of enzymes involved in the biosynthesis of flavonoid skeleton molecules. The basic structures of flavonoids are formed by a type III PKS, CHS. The broad substrate promiscuity and functional diversity of the type III PKSs may be a driving force for expanding the chemical variety of specialized metabolites. In other specialized metabolisms, isomerases (e.g., oxidosqualene synthase) and lyases (e.g., terpene synthase) are also involved in scaffold formation and may contribute the chemical diversity of secondary metabolites. Modification enzymes such as GTs (glycosyltransferases) and acyltransferases also contribute greatly to the huge diversity of flavonoids and other secondary metabolites. Interestingly, plants have two types of flavonoid GTs: the cytosolic family 1 GTs and the vacuolar glycoside hydrolase family 1 (GH1) (Cao et al., 2017). Acylation is also catalyzed by differentially localized enzymes: cytosolic BAHD acyltransferases and vacuolar SCPL acyltransferases, derived from serine carboxypeptidase (Milkowski and Strack, 2004; Moghe and Last, 2015). It is still unknown why plants have evolved these differentially localized enzymes for the modification of flavonoids and other specialized metabolites. The evolution of the family 1 GTs, the GH1s, and the BAHD acyltransferases in plants have been reviewed elsewhere (St-Pierre and De Luca, 2000; Yu et al., 2009; Tuominen et al., 2011; Yonekura-Sakakibara and Hanada, 2011; Caputi et al., 2012; Moghe and Last, 2015; Cao et al., 2017). Throughout their long history, plants have engineered their metabolic pathways to adapt themselves to their habitats and growth conditions in tissue and organ specific manners. A detailed understanding of the evolutionary history of metabolic enzymes involved in biosynthesis, modification, transport, secretion, transcriptional regulation, and chemodiversity will assist us in the engineering of specialized metabolic pathways to produce desirable metabolites with minimal energy expenditures.

## Author Contributions

KY-S proposed the concept. KY-S, YH, and RN developed it and wrote the manuscript.

### Conflict of Interest Statement

The authors declare that the research was conducted in the absence of any commercial or financial relationships that could be construed as a potential conflict of interest.
